# 

**Published:** 1897-09

**Authors:** 


### CONTENTS.

ORIGINAL ARTICLES: PAGE

The Typhoid Serum-Diagnosis. By H. L. BOLLEY, M.S. . . . . . . 543
A Review of the Field of Veterinary Science. By LEONARD PEARSON, BSc.,V.M.D. 551
Significance of the Caption of a Paper or an Address. By A. W. BITTING, D.V.M. 560
Some Salient Points in Canine Practice. By WALTER AMOS, V.S. . . . . . 562
Pathological Shoeing. By W. H. YINGST, V.S. . . . . . . . . . 567

SELECTIONS . . . . . . . . . . . . . . . . . . . 573

EDITORIALS:

The Veterinary Profession: Its Relation to the Health and Wealth of the Nation . 576

Alaska—Klondike . . . . . . . . . . . . . . 577

The Future of Serum-Therapy . . . . . . . . . . . . 577

Where Next? . . . . . . . . . . . . . . . 578

A Well merited Appointment . . . . . . . . . . . . 579

The Way of the Transgressor is Hard . . . . . . . . . . 579

Better Safeguards Needed . . . . . . . . . . . . 579

Well-deserved Recoguition . . . . . . . . . . . . 580

Should Now Receive Consideration . . . . . . . . . . 580

Marriages . . . . . . . . . . . . . . . . 581

REVIEWS:

Bulletin No. 64, of the Virginia Agricultural Experiment Station . . . . . 581
Department of Agriculture Reports. The Twelfth and Thirteenth Annual Reports of the Bureau of Animal Industry . . . . . . . . . . . 581
Exercises in Equine Surgery . . . . . . . . . . . . 582

CORRESPONDENCE . . . . . . . . . . . . . . . 583

CONTROL WORK . . . . . . . . . . . . . . . . 583

LEGISLATION:

California . . . . . . . . . . . . . . . . 586

EXAMINATION QUESTIONS: Ohio State Board of Examiners, 1897 . . . . 587

SOCIETY PROCEEDINGS:

Coming Meetings—Veterinary Association of the District of Columbia—Chicago Veterinary Society—Colorado State Veterinary Medical Association . . . 589-593

AMONG THE COLLEGES: University of the State of New York—Harvard—Louisiana State University—The United States College of Veterinary Surgeons—Veterinary Department, Columbian University . . . . . . . . . . . 595

KEEP YOURSELF POSTED ON CURRENT EVENTS . . . . . . . 596

PERSONALS . . . . . . . . . . . . . . . . . 601

NEW INVENTIONS . . . . . . . . . . . . . . . 604

